# Challenges and opportunities in examining and addressing intersectional stigma and health

**DOI:** 10.1186/s12916-018-1246-9

**Published:** 2019-02-15

**Authors:** Janet M. Turan, Melissa A. Elafros, Carmen H. Logie, Swagata Banik, Bulent Turan, Kaylee B. Crockett, Bernice Pescosolido, Sarah M. Murray

**Affiliations:** 1 Department of Health Care Organization and Policy, School of Public Health, University of Alabama at Birmingham, 1665 University Boulevard, Birmingham, AL 35294 USA; 2 Department of Neurology, Johns Hopkins Hospital, Baltimore, MD USA; 3 Factor-Inwentash Faculty of Social Work, Toronto, ON Canada; 4 Women’s College Research Institute, Women’s College Hospital, Toronto, ON Canada; 5 Department of Public Health & Prevention Sciences, Baldwin Wallace University, Berea, OH USA; 6 Department of Psychology, University of Alabama at Birmingham, Birmingham, AL USA; 7 Department of Sociology, Indiana University, Bloomington, IN USA; 8 Department of Mental Health, Johns Hopkins Bloomberg School of Public Health, Baltimore, MD USA

**Keywords:** Layered stigma, double stigma, multiple stigma, overlapping stigma, discrimination, prejudice, measurement, intersectional

## Abstract

**Background:**

‘Intersectional stigma’ is a concept that has emerged to characterize the convergence of multiple stigmatized identities within a person or group, and to address their joint effects on health and wellbeing. While enquiry into the intersections of race, class, and gender serves as the historical and theoretical basis for intersectional stigma, there is little consensus on how best to characterize and analyze intersectional stigma, or on how to design interventions to address this complex phenomenon. The purpose of this paper is to highlight existing intersectional stigma literature, identify gaps in our methods for studying and addressing intersectional stigma, provide examples illustrating promising analytical approaches, and elucidate priorities for future health research.

**Discussion:**

Evidence from the existing scientific literature, as well as the examples presented here, suggest that people in diverse settings experience intersecting forms of stigma that influence their mental and physical health and corresponding health behaviors. As different stigmas are often correlated and interrelated, the health impact of intersectional stigma is complex, generating a broad range of vulnerabilities and risks. Qualitative, quantitative, and mixed methods approaches are required to reduce the significant knowledge gaps that remain in our understanding of intersectional stigma, shared identity, and their effects on health.

**Conclusions:**

Stigmatized identities, while often analyzed in isolation, do not exist in a vacuum. Intersecting forms of stigma are a common reality, yet they remain poorly understood. The development of instruments and methods to better characterize the mechanisms and effects of intersectional stigma in relation to various health conditions around the globe is vital. Only then will healthcare providers, public health officials, and advocates be able to design health interventions that capitalize on the positive aspects of shared identity, while reducing the burden of stigma.

## Background

‘Intersectional stigma’ is a concept that has emerged in the literature to characterize the convergence of multiple stigmatized identities within a person or group and to address their effects [1]. An intersectional perspective allows researchers, health professionals, and advocates to think holistically about how living with multiple stigmatized identities affects behaviors, as well as individual and population health outcomes [2]. Intersectional approaches also highlight protective factors, such as social support, resistance, and adaptive coping strategies, that emerge when people with similar identities unite [3].

Researchers in sociology, political science, and economics have long examined how characteristics such as race, sex, and health status affect privilege and disadvantage at the individual and societal level. While individuals as far back as Sojourner Truth characterized the experiences of being a Black woman in societies that devalue both female gender and minority racial status [4, 5], the term intersectionality is largely attributed to Crenshaw’s 1989 work [5]. Since its introduction, intersectionality has transformed multiple fields of research. Instead of viewing characteristics like gender expression or poverty in isolation, intersectional theory codified efforts to examine these individual axes of difference in tandem [6]. It also encouraged researchers to critically examine how systems of oppression interact at the societal, community, and individual levels [7–10].

Stigmatization is a process rooted in the ways that society negatively views individual or group characteristics or identities. Building on sociologist Goffman’s seminal 1963 description of the social processes of labeling and social exclusion implicated in stigma [11], conceptualizations of stigma have developed to include socio-cognitive approaches that center on the psychological impacts of stigma (e.g., Herek [12]) and analyses of systems of power involved in (re)producing inequity (e.g., Link and Phelan [13]).

Health-related stigma is defined as “*a social process or related personal experience characterized by exclusion, rejection, blame, or devaluation that results from experience or reasonable anticipation of an adverse social judgment about a person or group identified with a particular health problem*” [14]. It encompasses both prejudicial attitudes, beliefs, and values as well as discriminatory behavior, practice, and policies. Substantial bodies of research indicate that stigma related to either health problems or identities adversely affects health. However, much of this literature focuses on the consequences of only one form of stigma in isolation. The need to recognize an individual’s membership in multiple stigmatized groups has been a relatively recent consideration in the public health literature [1, 15, 16]. This acknowledgement is largely due to efforts to understand HIV-related stigma not only as a manifestation of fears related to the health condition itself, but also negative attitudes regarding behaviors and identities originally associated with HIV transmission (e.g., sexual practices or orientation, injection drug use, and sex work) [10, 17].

An intersectional perspective is vital to understanding the experiences and consequences of living with multiple stigmatized identities. Rich bodies of research highlight the deleterious impacts of different forms of stigma and discrimination on health outcomes, and the nascent intersectional stigma research points to the ways in which multiple forms of health-related stigma are experienced and how their combined effects influence both healthcare access and outcomes. In what follows, we discuss intersecting stigmas that fall into three categories aligned with Goffman’s general categorization of stigma processes, namely (1) physical health ailments, Goffman’s “*abominations of the body*” (e.g., HIV, mental health issues, epilepsy, cancer, infertility); (2) affiliations with marginalized groups, Goffman’s “*tribal*” forms of stigma (e.g., racial or ethnic identity, gender, sexual orientation); and (3) factors attributed to one’s ‘moral’ character or behaviors, Goffman’s “*blemishes of individual character*” (e.g., smoking, alcohol use, substance use issues, sex work, incarceration, gender-based violence, abortion, obesity, poverty). While terms other than ‘intersectional stigma’ have been proposed, such as ‘layered’, ‘double’, ‘overlapping’, and ‘multilevel’ stigma, these terms all inadvertently imply mechanisms in addition to describing a theoretical approach. ‘Double stigma’, for example, implies that the experiences and effects of living with multiple stigmatized identities are simply additive (‘doubled’), when in fact they may be multiplicative or interact in other complicated ways to produce a given experience [18–20].

Given the complex identities and challenges facing people with acute and chronic health problems across the globe, it is imperative that health interventions consider the vulnerabilities and strengths of affected groups [7]. Interventions that deal solely with a single health-related stigma, without considering the co-experience of stigmas, marginalization, and resilience associated with other conditions, identities, or behaviors, are likely to have limited success in reducing health disparities and making lasting improvements in health. In this article, we summarize existing knowledge on intersectional stigma and health, identify gaps in knowledge and methods for studying and addressing intersectional stigma, and propose priorities to guide future health research. Our goal is to introduce clinical researchers to the variety of methods available for studying intersectional stigma and provide examples of how these methods can be used to examine intersectional stigma and health.

## Existing literature on intersectional stigma and health

Existing literature on intersectional stigma and health can roughly be divided into two categories, addressing (1) manifestations of intersectional stigma and (2) effects of intersectional stigma on health behaviors and outcomes. Within both categories, much of the existing research focuses on people living with HIV due to prioritization by the World Health Organization and National Institutes of Health. However, other stigmatized conditions, such as mental illness, epilepsy, and physical disabilities, have a long history from which we will draw to illustrate the range of intersectionality research.

### Manifestations of intersectional stigma

Intersectional theory has been used to examine manifestations of stigmatization from two perspectives, namely from those of the public and from stigmatized individuals. Public perceptions of stigma have largely been examined by measuring preferred social distance from individuals with stigmatized conditions via multi-item scales and/or vignettes. Participants are asked about their willingness to interact with the stigmatized individual in different contexts. Change in social distance associated with additional identities or conditions can be calculated using a singular stigmatized characteristic as a comparison. While this approach may underestimate the impact of stigmatization, since it infers behavioral responses from self-reported intentions and may be subject to social desirability bias [21], recent research suggest that individuals are less guarded in expressing negative attitudes than in actually discriminating in person [22].

Studies using this methodology have found that intersectional stigma is shaped both by views of how severely a given identity deviates from accepted social norms and the extent to which ‘victim blaming’ is associated with each identity [23]. For example, college undergraduates and healthcare providers presented with vignettes of individuals with HIV have repeatedly indicated greater social distancing when injection drug use or a gay identity is also associated with that individual [24–27]. In a general population study, individuals responding to a pregnant woman with opioid addiction endorsed lower stigma when vignettes depicted successful treatment, but only for high socioeconomic status women [28]. The effect of intersectional stigma appears dependent on the stigmatized characteristics involved. In a vignette-based study of stigmatization by undergraduate students, Walkup et al. [29] noted that the inclusion of HIV-positive status in descriptions of individuals did not substantially increase stigmatization related to mental health issues. This could be due to the highly negative connotation associated with conditions such as schizophrenia or to the dearth of quantitative measures sufficiently sensitive to assess intersectional stigma. Focusing on mental illness and social distance, Box 1 describes an approach to examining public perceptions of intersectional stigma where vignettes were used to describe individuals with schizophrenia and other stigmatized identities.

Existing literature indicates that experiences of intersectional stigma are dependent not only on the stigmatized traits present, but also on the characteristics associated with those traits. For example, Lekas et al. [30] found that New Yorkers with comorbid HIV and hepatitis C virus (HCV) reported greater HIV-related stigmatization than HCV-related stigma, yet some indicated that both conditions were equally stigmatized due to their association with injection drug use. While a comparison group of individuals with only HIV or HCV was not included, this suggests that intersectional stigma may be influenced by characteristics that are perceived, but not necessarily present. Similarly, while individuals with HIV and tuberculosis report greater HIV-related stigma than individuals with only HIV [31], this may have little to do with tuberculosis itself. Qualitative data suggest that tuberculosis-like symptoms have been interpreted by the public as a marker for a previously concealed HIV diagnosis [32, 33]. In sub-Saharan Africa, where both epilepsy and HIV continue to be highly stigmatized, Zambian adults with comorbid HIV and epilepsy were more likely to feel as though others blamed them for their HIV status [34]. Further, they reported greater feelings of depersonalization than adults living only with HIV and endorsed greater epilepsy-associated stigma than those with only epilepsy. Some research suggests that individuals who report stigma from one condition may be more likely to also endorse stigma due to a second condition, possibly due to their sensitivity to stigmatizing experiences [35].

Although stigmas have been found to exacerbate one another [24, 29], qualitative and quantitative research have shown that some stigmatized characteristics may even mitigate the stigma related to other characteristics [26, 36]. For example, in a national survey, Black American adults reported less internalized weight-related stigma compared to White adults [37]. Among HIV-positive Black women, increasing age was associated with decreased HIV-related stigma [38].

### Effects of intersectional stigma on health behaviors and outcomes

Intersectional stigma has been repeatedly associated with worse health behaviors and outcomes. Co-existing racial discrimination among African American adults living with HIV has been associated with decreased HIV disclosure [39] and worse medication adherence in the United States [40]. More severe symptoms of depression have been seen among HIV-positive men who reported increased stigma due to having sex with men in India [41], HIV-positive women and racial minorities reporting HIV or racial stigma in Canada [42], and persons living with comorbid HIV and tuberculosis infection in Lesotho [43]. Studies of transgender women indicate lower access to HIV-related healthcare relative to cisgender people, primarily due to pervasive transphobia in healthcare [44]. Lacombe-Duncan [45] argues that these disparities may be explained by intersecting systems of oppression associated with stigmatized identities. For example, the association between HIV-associated stigma and access to regular HIV care has been shown to be modified by multiple other stigmas, including substance use stigma, among Russians living with HIV who inject drugs [46] and sex work stigma among female sex workers in the Dominican Republic [47].

An intersectional approach has also helped illuminate how individuals cope with stigmatized identities. Individuals with comorbid HIV and HCV infection reported concealing their hepatitis status [30], just as professional dominatrixes conceal their roles from other commercial sex workers [48], to decrease encountered stigma. Finally, people in stigmatized groups may find solidarity within their community, which can offer protection against some of stigma’s negative effects. Among Black American women with HIV in Chicago, awareness of systemic oppression and a desire to join others to enact social change (‘critical consciousness’) was associated with a higher likelihood of a CD4 count greater than 350 and a lower likelihood of detectable HIV viral load when perceived racial discrimination was high [49].

## Measurement and analytical approaches for intersectional stigma

Intersectionality is a lens through which researchers seek to understand the complex nature of identity, health, social relationships, and power that plays out within human interaction and experiences. The ambiguities embedded in how to use intersectionality to understand the world make it a flexible tool that is popular across disciplines. At the same time, differences have led to debate about appropriate approaches and methods. Intersectionality is not prescriptive in its methods, and there is no consensus on what specific data collection and analysis methods are best suited for implementing research on this topic [6, 50]. Yet, applying intersectionality to the study of stigma and health requires methodological techniques that appropriately characterize complex relationships across multiple marginalized identities or stigmatized conditions.

McCall [50] summarized attempts to understand these differences into three approaches that have been expanded upon by others, namely anticategorical, intracategorical, and intercategorical. An anticategorical approach “*deconstructs*” categories that are seen to limit understanding through oversimplification (e.g., sexual practice categories that fail to acknowledge membership in multiple groups). An intracategorical approach is characterized by in-depth exploration into a particular constellation of identities and conditions (e.g., professional female dominatrices [48]). The third approach, intercategorical, allows comparisons between groups or individuals with different identities or experiences (e.g., Black men who have sex with men versus Latino men who have sex with men [51]). Due to the need for methods that can transcend categorization (anticategorical) and provide thick description (intracategorical), qualitative methods are often used when examining stigma and health. Increasingly, intersectional studies have also employed a variety of quantitative methods. In the following sections, we explore the application of qualitative and quantitative methods to the study of intersectional stigma and health. We aim not to prioritize one set of methods over another, but rather to encourage the use of a diversity of methods, the choice of which should be driven by the research question.

### Qualitative approaches

Qualitative methods, including data collected using in-depth interviews, focus groups, ethnography, photo voice, and observation, offer insight into what intersectional stigma individuals with multiple identities experience, particularly those who are often overlooked by health research. In addition to providing a perspective for policymakers and providers whose services are geared towards specific populations, qualitative research augments existing theory while generating new ideas and hypotheses regarding forms and mechanisms of stigmatization [18, 52]. Qualitative research methods can also be used to explore the relative experiences of different types of stigma. Recent qualitative intersectional studies have focused on stigma related to a variety of health conditions and behaviors, including HIV [2, 3, 53], tobacco use [54], and mental health [55], most commonly as they relate to racial identity, gender, socioeconomic status, and sexual orientation. While intersectionality theory has been used in studies with diverse qualitative methodologies, a recent HIV-related review found that intersectionality was rarely the central focus [56] (see [41, 55, 57] for exceptions). More commonly, intersectionality is used to explain findings that emerge from qualitative data using grounded theory [2, 3, 58], discourse analysis [54], a case narrative approach [55], and general thematic or inductive analysis [59, 60]. Qualitative methods are also often used in the context of community-based participatory research around intersectional stigma and other multidisciplinary approaches [61, 62]. Box 2 presents an example of the use of qualitative methods to examine intersectional stigma experienced by transgender women in India.

### Quantitative approaches

Quantitative methods address complimentary research questions, such as generating population-based prevalence estimates of stigma experiences, which are essential for demonstrating stigma burden and planning responsive services and interventions. Common quantitative methods, such as multinomial logistic or linear regression, overlook the complexity of co-existing stigmas by controlling for factors such as race, gender, or class that may shape the way stigma is experienced. While the simplistic approach is to treat their effects as additive [63], researchers are increasingly using non-additive approaches to quantitative modeling that allow more flexibility in demonstrating how co-existing stigmas interact to shape outcomes.

### Measurement and instruments

A common approach to measuring intersectional stigma involves asking parallel questions on stigma and discrimination related to different identities. An example is the Everyday Discrimination Scale [64], in which individuals are asked about the same experiences in reference to their race, economic situation, HIV status, etc.; this approach is illustrated in an example from the Deep South in the United States in Box 3. A second approach is measuring stigma related to one identity or health condition, then examining how that stigma experience varies among individuals according to membership in various stigmatized subgroups. Because parallel questions or a single instrument may not appropriately capture the nuances of different stigmas, a third approach requires the use of a condition-specific measure for each stigma studied. This approach is illustrated in an example from Jamaica, presented in Box 4. Quantitative instruments can also be used to assess unique experiences of intersectional stigma within a specific group, such as Rosenthal and Lobel’s [65] work examining gendered racism among Black and Latina women in the United States. Determining to what extent stigma varies across conditions and identities is a key consideration when deciding whether condition-specific or general measures of stigma should be used in intersectional stigma research and program evaluation.

### Analytical strategies

Table 1 summarizes the methodological approaches for characterizing intersectional stigma. Herein, we discuss some of the most commonly used strategies, as well as less frequently employed methods that address key limitations of other approaches.

**Table 1:** Overview of quantitative analysis strategies for modeling intersectional stigma

Strategy	Description	Advantages	Limitations	Examples	Recommendations for use
Stratified analyses	The relationship between a measure of stigma and a health outcome is analyzed in separate samples disaggregated by an identity of interest (e.g., illness status, gender, race)	• Simple, easy to perform and interpret	• Cannot necessarily test for statistical significance [101] • Difficult to interpret and cumbersome to perform when multiple axes are considered • Cannot use for two continuous measures of health-related stigma	• Exploration of educational outcomes among individuals of Mexican origin only within a sample of women [102]	• Exploratory questions about how the relationship between stigma and health might vary in the presence of an additional identity-related factor that is discrete (e.g., HIV-status, gender)
Factorial design	Vignettes that describe individuals with different combinations of characteristics or identities are presented, typically randomly, to a general population sample. Individuals’ responses to a measure of stigma (e.g., social distance) across vignettes are compared [103]	• Allows for decomposition of stigma related to different identities or factors into unique and shared components [103] • Experimental design	• Can reflect additive assumptions about the nature of intersectional stigma [52] • Difficult to interpret and cumbersome to perform when multiple axes are considered • Difficult to include explanatory or process variables [52]	• Disentanglement of stigma associated with HIV from stigma associated with risk practices (e.g., injection drug use) [104]	• How the level of community discrimination or stigmatizing attitudes and beliefs may vary based on the presence or absence of a small number of additional behavioral or identity-related factors
Moderation analysis	The main effects of two (or more) stigma-related variables are modeled along with the product of those variables (e.g., race × gender × HIV status)	• Simple to do, and in the case of two-way interactions, to interpret • Flexible • Can assess positive or negative changes in magnitude and directionality of effects [63]	• When main effects explain much of the variance in the outcome, the ability to assess interactions between those terms is limited [20] • Three-way or higher-order interactions are difficult to depict and comprehend	• Examination of how social adversity, HIV status, and race interact to explain depression [105] • Assessment of how stigma related to HIV and substance use interact to explain depression [106] • Assessment of how weight discrimination interacts with race and socioeconomic status to shape mental health among women [107]	• When large sample sizes are available and variation is present within subgroups to test how the relationship between stigma and health might vary in the presence of an additional identity-related factor that is discrete (e.g., HIV-status, gender)
Latent class or latent profile analysis	Identifies subpopulations of individuals based on their endorsement of different stigma or discrimination experiences Predictors of membership in these populations (such as identity characteristics like race or health status) can be evaluated and latent class regression can be used to assess how these different patterns of experiences differentially predict health outcomes	• A person-centered, rather than variable-centered, approach to assessing intersectionality • Treats different patterns of stigma experiences as latent and allows these to be empirically determined	• Can require large sample sizes • More difficult to explain to lay audiences, including policymakers and funders in some cases	• Identification of patterns of bullying and discrimination experiences related to different identities and assessed to what extent these patterns differentially predicted mental health outcomes [73]	• When large sample sizes are available and the question of interest is how the nature of stigma may vary based on the presence of different combinations of stigmatized behaviors or identities
Multilevel models	In addition to fixed effects, random effects (intercepts and slopes) at the cluster level (e.g., neighborhood, city, country) are included in regression models Covariates can be included at both levels of analyses and cross-level interactions can be modeled	• Enables modeling of structural level influences on stigma and health • Can be used for analysis of intensive longitudinal data by accounting for correlation of observations within person over time	• More difficult to explain to lay audiences, including policymakers and funders in some cases • Requires data collection in multiple contexts and, in some cases, may require existing data at higher levels (e.g., state or country level data)	• Exploration of whether the relationship of gender, class, and race to self-rated health varied by neighborhood [63] • Assessment of how country-level and individual level factors interact to influence the mental health of male sexual minority European migrants [19] • Examination of how everyday experiences of discrimination impact internalized stigma among people living with HIV using a smartphone-based experience sampling method survey [64]	• When multiple time points are available or data is available from multiple clusters (the number necessary will vary, but 10–15 would be considered few clusters for an analysis [108]) and contextual influences on the relationship between stigma and health are of interest
Structural equation modeling	Allows for simultaneous estimation of measurement and structural components, including pathways between observed and latent variables	• Appropriately models measurement error associated with inclusion of latent variables • Flexible strategy: can simultaneously assess the impact of multiple exposures on multiple outcomes, include group-based or multilevel modeling, and assess moderated mediation or mediated moderation [109] • Can assess how exposures and outcomes predict each other over time	• Modeled relationships may be inappropriately interpreted as causal • Depending on the number of parameters included, may require larger sample sizes to be estimable • Not all models may be identifiable and sensitive to model misspecifications	• Simultaneous assessment of experiences of racial discrimination and HIV-related stigma on quality of life among African and Caribbean Black women in Canada [74]	• For estimating complex models including multiple stigma-related factors as predictors or multiple related health outcomes of interest, particularly when including psychosocial variables that are not directly observable (e.g., stress, coping)

#### Moderation approaches

Statistical moderation occurs when the effect of one variable on an outcome depends on the level of a second exposure variable [66]. Including an interaction term as part of a quantitative model allows researchers to assess the main effects of two stigma-related variables (*A* and *B*), as well as the extent to which the effect of one variable is moderated by the other (denoted *AxB*). This widely applicable method has become the most commonly used analytical strategy for modeling intersectional stigma. An advantage to this approach is its flexibility; interactions can be included in a variety of models (e.g., linear, Poisson, or logistic regression) and can be generated between varying numbers of factors (e.g., two-way, three-way interactions). Further, interaction terms are not limited by the type of variable included. Factors that are typically treated as categorical, such as gender, and more nuanced continuous measures, such as stigma severity, can be used to generate interaction terms. Interaction terms can model the conjoined effects of a health-related stigma with an identity variable, two identity variables, or two health-related stigmas. If the interaction term is significant, follow-up analyses are conducted to reveal the nature of the interaction [67]. While a useful tool, interaction terms have limitations in application to the study of intersectional stigma. For instance, when main effects explain a large amount of the variance in an outcome, it can be difficult to detect small but meaningful interactions between these variables and other stigmatized characteristics.

#### Multilevel modeling

Also known as nested or hierarchical models, multilevel models allow the effect of independent variables, such as gender and class, to vary by individual or group [66]. This allows for better characterization of the social context in which identities affect health [18, 51]. Pachankis et al. [19] used multilevel regression to characterize how macro- and individual level factors intersect to shape HIV-risk for European migrant men who have sex with men. By including individual-level covariates (e.g., origin and country of immigration) and country-level factors (e.g., national laws and attitudes), they found that both anti-gay and anti-immigrant policies in the country of immigration affected individual HIV risk [68]. They also found that the effects of country-level factors were moderated by individual characteristics such as duration of residence in the country of immigration (i.e., cross-level interactions) [19]. Multilevel frameworks can be used in studies employing ecological momentary assessment (also known as experience sampling) to evaluate the impact of real-time intrapersonal experiences of intersectional stigma measured at several time points on individual health outcomes and behaviors [69, 70]. A challenge with multi-level modeling is the ability to collect data across enough contexts to assess the effects of second level factors.

#### Latent variable, latent class, and latent profile methods

Latent variable methods allow researchers to examine traits that cannot be directly measured, but can be inferred from other directly assessed characteristics (e.g., measuring depression via self-report of symptoms) [71]. Latent class and latent profile analysis (where indicators are categorical or continuous, respectively) allow researchers to assess whether measured identity or health-related variables predict an individual’s membership in an inferred group. Unlike traditional variable-centered, additive models, where stigmas are assumed to affect outcomes in the same way for everyone, latent class analysis takes a person-centered approach by identifying subgroups of individuals based on their stigma experiences and how those patterns of stigma experiences shape outcomes [72]. While it is often problematic in other methodologies to ask individuals to identify which personal characteristics cause them to experience discrimination, because people often do not know which aspect(s) of their identity is related to the way they have been treated [20], this approach overcomes this challenge by treating stigma as a holistic embodied experience. Latent class analysis allows researchers to identify subgroups of people with different patterns or profiles of stigma, investigate whether outcomes vary across these groups, and assess whether a given stigmatized characteristic or identity predicts membership in groups defined by different stigma experiences. Garnett et al. [73] used this approach to identify four patterns of discrimination and bullying among adolescents based on race, immigration status, weight, and sexual orientation. One of the subgroups identified was an intersectional class characterized by high probabilities of bullying and both weight- and race-related discrimination. While membership in all but the low discrimination class was associated with depressive symptoms, only membership in this intersectional class was associated with higher odds of suicidal ideation [73]. This study and others were able to identify subgroupings of individuals based on discrimination experiences, which has direct implications for being able to effectively target interventions [1]. However, a sole focus on experiences of discrimination may ignore the way that other facets of stigma (such as internalized stigma) may also shape individuals’ health.

#### Structural equation modeling (SEM)

SEM is a data analysis method that allows pathways and relationships to be estimated among observed and latent variables by allowing for simultaneous estimation of measurement and structural components. In addition to better accounting for measurement error in constructs that are not directly observable, another strength of SEM is the flexibility to measure complex interrelationships between multiple health outcomes, different forms of stigma, and other risk or protective factors. By using this approach to assess stigma related to HIV and racial discrimination among women living with HIV in Canada, Logie et al. [74] determined that depression and low social support mediated the effects of these experiences on quality of life, but also that racial discrimination was independently associated with HIV-related stigma (Box 5).

### Mixed methods approaches

The ideal methodological approach combines both qualitative and quantitative approaches, as this allows quantitative research to be more grounded in the lived experiences of people [18], while ensuring that aspects of stigma that emerge at the intersections of identities are measured in testable ways. When conducting quantitative intersectional stigma research, a researcher must decide which identities, behaviors, or health conditions receive attention in analyses, since not all combinations make sense or are of equal importance [18, 75]. A mixed methods approach guides these choices, resulting in more statistical power and less superfluous testing. This approach also provides needed population-based estimates of stigma burden across conditions, data on the effectiveness of different intervention strategies, and an in-depth understanding of why and how interventions should address intersectional stigma [18, 20]. To be successful, mixed methods approaches require research teams that work across disciplines with skills in different analytical approaches.

## Discussion and future directions

People experience intersecting forms of stigma that influence health behaviors, as well as their mental and physical health. Different stigmas are often correlated and interrelated, and their combined effects can be additive but may often be more complex. An intersectional approach can be useful in guiding the interpretation of findings on stigma and health, whether qualitative or quantitative [61]. However, significant gaps in our understanding of intersectional stigma must be addressed to improve individual and population health outcomes.

As shown in Box 6, the following areas should be prioritized to move this field forward. Firstly, more valid and reliable ways of measuring and analyzing data on intersectional stigma are required. This will allow a more thorough investigation of the impact of intersectional stigma on health outcomes, as well as examination of associated mechanisms and longitudinal effects. Secondly, the drivers of intersectional stigma, as well as the interpersonal, psychological, and biological mechanisms for effects on health outcomes, require additional elucidation. Drivers and mechanisms of intersectional stigma are not necessarily different from those of single stigmas, but may be more complex when they occur simultaneously. Further investigation into how the experience of intersectional stigma changes based on health condition and setting is warranted. Qualitative data suggest that intersectionality may have different salience based on historical, cultural, and socioeconomic contexts. The intersection of health-related and racial identity stigma, for example, may be more salient in the United States or South Africa, where there are long histories of institutionalized discrimination (i.e., Jim Crow laws and apartheid), as compared to countries without such history. It is currently unclear whether certain types or combinations of stigma are more impactful on health behaviors and outcomes than others. Characteristics associated with increased health-related stigma, such as blame, concealability, perception of risk, and availability of treatment, may not have the same effect on behaviors and outcomes in the setting of intersectional stigma. It is also unknown how shifts in one type of stigma, such as HIV-related stigma, may affect stigma experienced in another dimension, such as transgender stigma, or the intersection of the two stigmas. Thirdly, the field needs to better characterize the potential positive effects of shared identity so that these may be harnessed to improve health outcomes and behaviors. Finally, intervention research is needed to address the barriers posed by intersectionality, as well as to capitalize on the solidarity and social support that people with similar identities share. Some HIV-related stigma-reduction interventions have already begun incorporating an understanding of intersectional stigma into their content [76, 77]. The goal of this program of research should be to identify groups that are especially in need of supportive interventions and to provide guidance on designing the most effective interventions. Further, when allocating resources to stigma research or stigma-reduction interventions, funders should consider intersectional approaches to help increase the impact of investments. Likewise, when appropriate, policymakers should prioritize stigma-reduction policies that consider multiple intersecting stigmas to maximize improved health outcomes. 

## Conclusions

While often examined in isolation, stigmatized identities do not exist in a vacuum. Most people experience intersecting forms of stigma, which have complex effects on health behaviors, physical health, and mental health. Intersectionality is an emerging approach to stigma research that can be used to better understand the experiences of vulnerable groups with multiple stigmatized identities, while providing guidance on intervention strategies that can reduce stigma, increase resilience, and improve health.

**Fig. 1:**
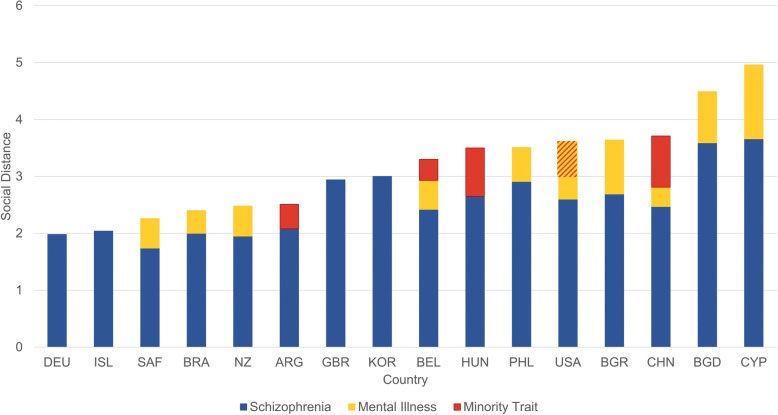
Preference for social distance due to schizophrenia and other minority traits (Box 1).

Box 1 Intersectional stigma and mental health in 17 countries Until recently, it was thought that traditional, community-wide ties in low- and middle-income countries led to greater acceptance of differences, resulting in decreased stigmatization. However, the Stigma in Global Content – Mental Health Study (SGC-MHS) has not found a consistent relationship between level of development and stigma [78–80]. The SGC-MHS conducted face-to-face interviews with nationally representative samples of adults on every continent. Participants were provided a vignette describing an individual with schizophrenia and asked about willingness to interact with that person across six social settings, including work, neighborhood, and marriage into the family. Vignettes randomly varied characteristics. including a second stigmatized condition that constituted an ‘outgroup’ (e.g., race/ethnicity/region) in that society (details at: www.indiana.edu/~icmhsr/sgcmhs.html). Participants were asked, among other things, to identify the condition as labeling has been shown to affect stigma. A social distance score was then created enumerating participants’ willingness to interact with the individual described in the vignette and examined based on characteristics presented. Results suggest that stigma from the public varies dramatically across countries and is affected by the presence of other devalued characteristics. As shown in Fig. 1, schizophrenia is highly stigmatized worldwide (blue). Inclusion of an outgroup (red) did not increase social distance among participants in Germany (DEU), Iceland (ISL), Great Britain (GBR), and South Korea (KOR). However, outgroup status did increase stigma among participants from Argentina (ARG), Hungry (HUN), and China (CHN). Labeling schizophrenia as a mental health issue (yellow) also significantly increased social distance among participants in most countries, including South Africa (SAF), Brazil (BRA), New Zealand (NZ), Bulgaria (BGR), the Philippines (PHL), Bangladesh (BGD), and Cyprus (CYP). In Belgium (BEL) and CHN, both labeling and holding minority group status increased stigma. Interestingly, among USA participants, labelling schizophrenia as a mental health issue significantly added to social distance yet minority group status decreased it.

Box 2 Tackling transphobia among healthcare providers in India Transgender women (TW), including *hijras*, who are constitutionally recognized as a third gender in India, experience a disproportionate HIV burden [81, 82]. Studies have documented lower HIV testing and care among sexual and gender minority individuals in India [83, 84]. TW often avoid accessing healthcare, despite a free public system, to avoid a perceived risk of stigma and unfair treatment by providers [84]. To address the sources of stigma in healthcare settings, understand what drives discriminatory attitudes and practices among providers, and learn how they may begin to be addressed, we undertook a three-phase study using exploratory, descriptive, and contextual qualitative approaches based on grounded theory [85]. We conducted focus group discussions and in-depth interviews with both TW (*n* = 42) and healthcare providers (*n* = 48). An emergent approach [86] allowed for a deeper understanding of the lived experiences of TW and provider perceptions. Findings suggested that female providers displayed more accepting attitudes toward TW compared to male counterparts. Influences on providers’ stigmatizing attitudes included intersectional stigma around sex work, drug use, and gender non-conformity, assumptions of sexual pathology/abnormality, fears of HIV transmission, and a lack of cultural and clinical competency. Five themes emerged as influencing providers’ treatment of TW patients, namely (1) knowledge of TW health issues; (2) attitudes towards TW; (3) competency in treating TW individuals; (4) willingness to provide services to TW; and (5) fear of courtesy stigma (stigmatization due to contact with a stigmatized population). Findings suggest that positive social contact between the two groups may decrease manifestations of intersectional stigma while building empathy, thus a cognitive behavioral intervention based on these findings is being implemented.

Box 3 Effects of HIV, race, and sexual orientation discrimination on depression in Alabama The Deep South region of the United States has the highest rates of HIV diagnoses and mortality in the nation [87]. Social conservatism that characterizes much of the South may perpetuate HIV, race, and sexual orientation-related stigmas that marginalize people at risk for and living with HIV. In this study, 203 people living with HIV who were on treatment and not using substances were recruited from an outpatient HIV clinic in Birmingham, Alabama (62% male, 60% Black, 51% gay or bisexual). Experiences of discrimination related to HIV, sexual orientation, and race were assessed using nine parallel items [64]. Findings suggest that HIV discrimination was positively associated with racial (*r* = 0.366) and sexual orientation (*r* = 0.258) discrimination. Sexual orientation discrimination was also associated with racial discrimination (*r* = 0.466). In a linear regression model with all three types of discrimination entered simultaneously, controlling for age, sex, race, and sexual orientation, HIV discrimination was positively associated with depression (*B(SE)* = – 2.83(0.50), *p* < 0.001, 95% CI 1.83 to 3.82); sexual orientation discrimination was negatively associated with depression (*B(SE)* = – 1.38(0.51), *p* = 0.01, 95% CI (– 2.38 to – 0.38); and racial discrimination was not significantly associated with depression (*B* = 0.43, *p* = 0.24)). In moderation analyses with the same covariates, the effect of HIV discrimination on depression was dampened when people endorsed experiences of sexual orientation discrimination (interaction effect: *B(SE)* = – 2.25(0.83), *p* = 0.01, 95% CI – 3.88 to – 0.61), suggesting different forms of discrimination are non-additive. HIV discrimination had a greater effect on depression scores in the absence of sexual orientation discrimination. This moderating effect was not observed with racial discrimination. Experiences with sexual orientation discrimination may build resilience to HIV discrimination in the South and certain identities may create community solidarity protective against depression.

Box 4 HIV-related stigma, sexual and gender identity stigma, and depressive symptoms among lesbian, gay, bisexual, and transgender (LGBT) persons in Jamaica HIV prevalence rates of 14–30% have been reported among gay, bisexual, and other men who have sex with men (MSM) in Jamaica [88, 89], which are far higher than the general population prevalence of 1.7% [90]. Recent work has also revealed high HIV prevalence – nearly 25% – among transgender women in Jamaica [91]. Stigma and violence targeting LGBT persons in Jamaica is reportedly pervasive [92–94]. In Jamaica, this stigma is institutionally sanctioned by the criminalization of same-sex practices among men and a lack of human rights protection for LGBT persons [95]. This study involved a community-based research project with Jamaica AIDS Support for Life. A total of 911 LGBT participants were recruited using chain referral sampling in Kingston, Montego Bay, Ocho Rios, and surrounding areas. Inclusion criteria were being above 18 years of age and identifying as gay, bisexual, or a MSM; lesbian, bisexual, or a woman who has sex with women; and/or transgender. Nearly two-thirds of participants (*n* = 569, 62.46%) identified as gay or bisexual men, or MSM; 22.05% (*n* = 205) identified as lesbian or bisexual women, or as women who has sex with women; and 15.04% (*n* = 137) identified as transgender women. This analysis included 439 participants who reported that they perceived themselves at medium or high risk of HIV infection. Perceived and enacted sexual and gender identity stigma were measured using an adapted version of Diaz et al.’s [96] Homophobia scale. HIV-related stigma was measured using Steward et al.’s [97] 10-item perceived stigma subscale. Depressive symptoms in the past 2 weeks were measured continuously using the Patient Health Questionnaire-2 [98]. HIV-related stigma was positively correlated with sexual/gender identity stigma (r = 0.446, *p* < 0.001). Sociodemographic factors associated with higher depressive symptoms included younger age, lower education level, area of residence, and greater food and housing insecurity. Linear regression modelling was conducted to examine the associations of HIV-related stigma and sexual/gender identity stigma (entered simultaneously) with depressive symptoms, controlling for age, monthly income, education level, living area, food insecurity, housing insecurity, and sexual and gender minority identity. Both HIV-related stigma (b = 0.009, 95% CI 0.001 to 0.018) and sexual/gender identity stigma (b = 0.031, 95% CI 0.009 to 0.053) were associated with higher depressive symptoms. No significant interactions were found between HIV-related stigma and sexual/gender identity stigma. Although HIV-related stigma is associated with stigma targeting sexual and gender identity, the lack of a significant interaction suggests that both stigmas have unique effects on depression. The lack of interaction between the two stigma types suggests the effects are additive in this example.

Box 5 Structural equation modeling to assess the impact of racial discrimination and HIV-related stigma on the well-being of African and Caribbean Black women living with HIV [99] Logie et al. [99] used structural equation modeling to operationalize their conceptual model created based on Link and Phelan’s fundamental cause theory for determinants of well-being [100] among African and Caribbean Black women living with HIV in Ontario. To understand how racial discrimination, HIV-related stigma, and housing insecurity collectively influenced depression, social support, and self-rated health, the researchers conducted a five-city cross-sectional survey among a purposive sample of women recruited through community organizations and health centers. The final analytical sample consisted of 157 adult African and Caribbean Black women. A structural equation model was estimated that included two latent variables (HIV-related stigma and social support) and four observed variables (depression, racial discrimination, housing insecurity, and self-rated health). The authors found that higher racial discrimination scores were associated with greater report of HIV-related stigma. While racial discrimination had a direct effect on depression and social support, its impact on self-rated health was mediated by the experience of HIV-related stigma. While longitudinal research on these pathways is an important next step, the use of SEM and simultaneous estimation of associations between variables in the models allowed the authors to better understand the way stigma related to different identities (such as ethno-racial identity and HIV status) and socioeconomic status interact to influence health.

Box 6 Recommendations and priorities for intersectional stigma and health **Measurement**

- Further development of quantitative and qualitative tools for measuring/understanding intersectional stigma, including (but not limited to):
  - ○ Quantitative measures that capture complex and unique intersectional experiences for specific populations and health conditions.
  - ○ Valid parallel questionnaire measures that can capture the common elements of intersectional stigma across populations and health conditions.
  - ○ Qualitative interview/focus group guides that stimulate participants to explain and reflect on their experiences of intersectional stigma.

**Effects**

- Examination of how the effects of stigma change for different health conditions.
  - ○ *Example research question: How do the effects of TB-related stigma differ from the effects of HIV-related stigma in influencing access to healthcare?*
- Elucidation of how characteristics associated with stigmas (blame, concealability, perception of risk, etc.) change in the setting of intersectional stigma.
  - ○ *Example research question: How does blame related to mental health stigma change according to whether the person is in a marginalized ethnic group?*
- Characterization of how shifts in one type of stigma affect the burden of other stigmas.
  - ○ *Example research question: How does reducing stigma around HIV in a community affect experiences of substance use stigma?*
- Examination of how experiences and effects of intersectional stigma change based on historical, cultural, and socioeconomic context.
  - ○ *Example research question: How do experiences of weight-related stigma and poverty stigma differ in impoverished settings versus high-resource settings?*
- Characterization of potential positive effects of shared identity.
  - ○ *Example research question: How does social support from people with similar intersectional identities change the way people react to and deal with stigma?*

**Drivers and mechanisms:**

- Elucidation of drivers of intersectional stigma.
  - ○ *Example research question: Are there common drivers of some co-occurring stigmas?*
- Characterization of the interpersonal, psychological, and biological mechanisms for the effects of intersectional stigmas on health outcomes.
  - ○ *Example research question: What are the pathways through which intersectional stigmas around cancer and race affect access to cancer treatment?*
- Identification of the most salient pathways that can potentially be addressed in intersectional stigma interventions.
  - ○ *Example research question: Is addressing mental health effects related to experiencing both sexual orientation- and HIV-related stigma a potentially effective way to improve health outcomes for men who have sex with men living with HIV?*

**Interventions**

- Developing strategies that address the barriers posed by intersectionality, while capitalizing on solidarity and social support of shared identities.
- Identifying what types of stigma are best addressed simultaneously/together in interventions.
- Deriving strategies that can be used to meaningfully and genuinely engage the people at the center of intersectional stigmas in the development of interventions.

**Policy and practice**

- Funders should consider intersectional approaches to maximize the impact of investments.
- Policymakers should prioritize stigma reduction policies that consider multiple intersecting stigmas, when appropriate.
